# Pigment-dispersing factor neuropeptides act as multifunctional hormones and modulators in tardigrades

**DOI:** 10.1098/rsob.240242

**Published:** 2025-03-05

**Authors:** Soumi Dutta, Lars Hering, Milena M. Grollmann, Niklas Metzendorf, Vladimir Gross, Kazuharu Arakawa, Susanne Neupert, Monika Stengl, Friedrich W. Herberg, Georg Mayer

**Affiliations:** ^1^ Department of Zoology, University of Kassel, Kassel, Germany; ^2^ Graduate School "Multiscale Clocks", University of Kassel, Kassel, Germany; ^3^ Central Coordination Office, BMBF Research Initiative for the Conservation of Biodiversity (FEdA), Senckenberg – Leibniz Institution for Biodiversity and Earth System Research, Frankfurt am Main, Germany; ^4^ Institute for Advanced Biosciences, Keio University, Tsuruoka, Yamagata, Japan; ^5^ Department of Animal Physiology/Neuroethology, University of Kassel, Kassel, Germany; ^6^ Department of Biochemistry, University of Kassel, Kassel, Germany

**Keywords:** *Hypsibius exemplaris*, Tardigrada, G protein-coupled receptor, nervous system, neuromodulator, gene expression

## Abstract

Pigment-dispersing factors (PDFs) are neuropeptides that play key roles in controlling the circadian rhythms in various insects, whereas their function remains elusive in other protostomes including tardigrades (water bears). Here we show that the three PDFs of the tardigrade *Hypsibius exemplaris* are co-localized in two pairs of inner lobe cells in the brain, whereas only one PDF occurs in four additional cerebral and two extracerebral cells. The axons of the inner lobe cells pass through the contralateral brain hemisphere, descend to the ventral nerve cord and terminate in two pairs of potential release sites in the posteriormost trunk ganglion. Using *in vitro* assays, we demonstrate that all three PDFs and their deorphanized receptor (PDFR) are functional. Widespread localization of PDFR suggests that tardigrade PDFs may act as multifunctional hormones and neuromodulators that control major functions including light detection, neural processing, locomotion, feeding, digestion, osmoregulation, growth, embryonic development and oogenesis/reproduction.

**Figure:**
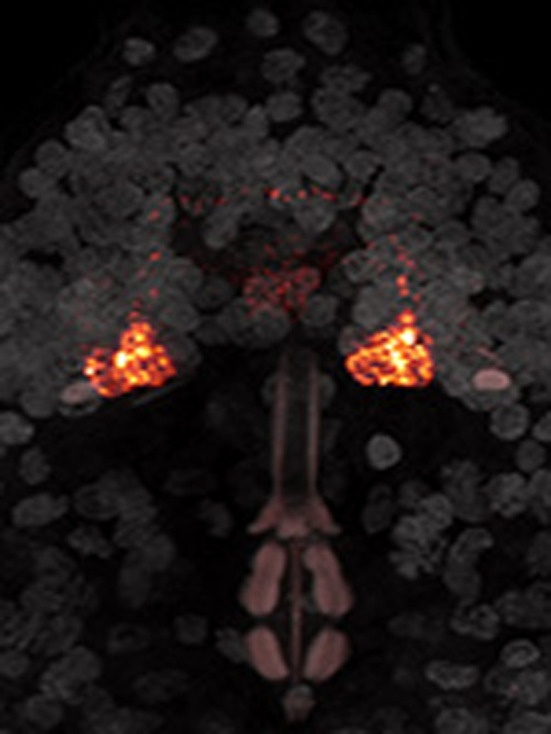

## 1. Introduction

**Figure 1.**
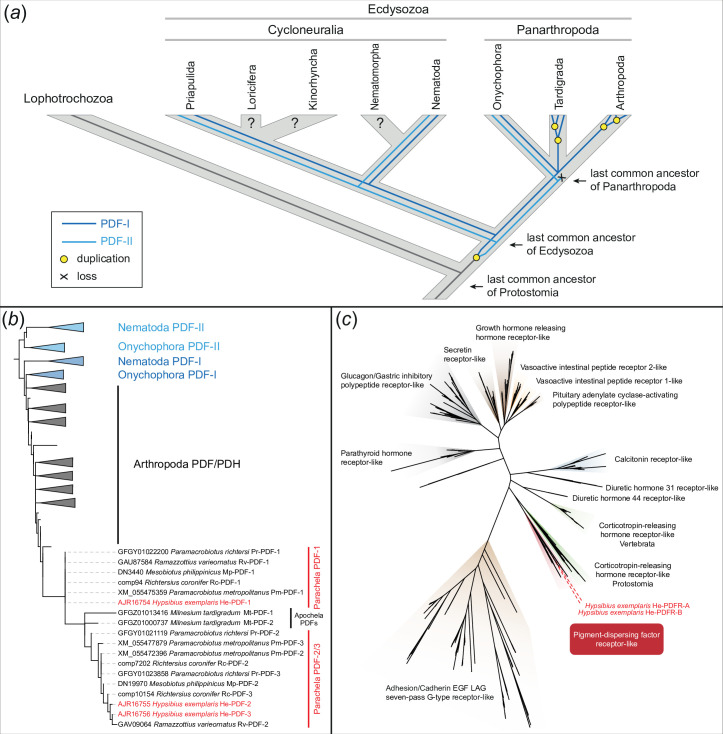
Evolutionary history of PDF/PDH peptides in ecdysozoans and phylogeny of PDF peptides and PDF receptor proteins. (*a*) Scenario on evolution of PDF peptides in ecdysozoans. Note the presence of two PDF peptides in the last common ancestors of Panarthropoda and Ecdysozoa. Modified from Mayer *et al*. [21]. (*b*) Maximum likelihood tree of PDFs across ecdysozoans. Note the position of monophyletic eutardigrade PDFs nested within the arthropod PDF/PDH clade and the occurrence of two distinct clades of parachelan PDFs (PDF-1 and PDF-2/3). PDF peptides of *H. exemplaris* are highlighted in red. (*c*) Maximum likelihood tree of approximately 1000 class B G protein-coupled receptor proteins (GPCRs). The dataset was obtained previously [20] from cluster analysis of approximately 18 000 bilaterian GPCRs. Both PDFR splice variants (He-PDFR-A and He-PDFR-B) of *H. exemplaris* fall into the monophyletic group of PDF receptors of protostomes (highlighted in red).

Pigment-dispersing factors (PDFs) are conserved neuropeptides that were initially identified in crustaceans as hormones (PDHs) that control epidermal colour changes and visual adaptation to light [1–3]. Subsequent studies suggested that PDFs play key roles in regulating the rest–activity rhythms in insects [4–12]. Besides their roles as clock output factors, they work as coupling factors between different clock neurons [13,14] and directly connect the bilaterally symmetric clock centres in both brain hemispheres [4–7,15]. They additionally control infradian rhythms of photoperiodic responses in different insects [16,17]. The circadian functions of PDFs in insects resemble those of the vasoactive intestinal peptide (VIP) in vertebrates [18,19], although the *pdf* and *vip* lineages are not sister groups and the ancestral *pdf* gene most likely evolved in protostomes [20]. While the last common ancestor of protostomes possessed only one *pdf* gene, a duplication might have led to two homologues in the last common ancestor of ecdysozoans (molting animals), followed by a subsequent loss of one of them in tardigrades (water bears) and arthropods (figure 1*a*). Repeated duplications likely gave rise to multiple *pdf* copies in tardigrades and decapod crustaceans [21].

Most information about the localization of PDFs is available from insects [4–11,13,16,17,22,23] but there are also some data from crustaceans [24–27], onychophorans (velvet worms) [20,21], nematodes [28] and molluscs [29]. The PDF-immunoreactive (PDF*-ir*) cells of insects are arranged in small clusters associated with the optic lobe neuropils of the compound eyes [4–6,8,10,11,13,16,30] and additional somata occur in the protocerebrum of some species studied to date [16,31,32]. PDF*-ir* neurons associated with the visual system arborize in a small neuropil, the accessory medulla, which was identified as a pacemaker centre controlling circadian locomotor activity rhythms [5,33]. A similar arrangement has been reported from crustaceans, but the existence of accessory medullae in this group and their involvement in the circadian clock have not been demonstrated [25,26,34].

While data on PDF immunoreactivity are unavailable from other arthropods, such as chelicerates (spiders and allies) and myriapods (e.g. centipedes and millipedes), there is no evidence of accessory medullae in onychophorans [20,21,35], which along with tardigrades represent the closest living relatives of arthropods (figure 1*a*). Hence, there are no PDF*-ir* somata directly associated with the onychophoran eyes that are most likely homologous with the median ocelli rather than the compound eyes of arthropods [35]. Despite the lack of accompanying PDF-*ir* somata, the visual neuropil of each onychophoran eye does contain numerous PDF-I*-ir* and PDF-II*-ir* fibres [20], most likely originating from a specific subset of protocerebral neurons. Besides in the protocerebrum, numerous PDF-*ir* somata occur throughout the remaining central nervous system of onychophorans, including the deutocerebrum, the circumpharyngeal nerve cords and the ventral nerve cords [20,21]. All PDF-*ir* neurons of onychophorans invariably contain both peptides, PDF-I and PDF-II, though these seem to be expressed at different levels in different groups of neurons [20].

Like onychophorans, the nematode *Caenorhabditis elegans* exhibits both ancestral *pdf* genes [21], *pdf-I* and *pdf-II* (figure 1*a*), whose products are localized in different neurons including sensory, motor, mechanosensory and interneurons [28]. Beyond this, both peptides occur in tissues and cells outside the nervous system, including those of rectal glands, the intestino-rectal valve and the arcade cells associated with the pharynx [28]. However, at least in the nervous system of *Caenorhabditis elegans*, PDF-I and PDF-II are not entirely co-localized [28]. Interestingly, the PDF receptor (PDFR) of nematodes is expressed in several tissues, suggesting that PDF-I and PDF-II may act as hormones that control multiple functions in these animals, including locomotion, chemosensation, mechanosensation and integration of other external stimuli [28,36–38]. Similar observations have been made in onychophorans, in which at least some PDFR-*ir* and PDF-*ir* cells are spatially separated [20] and there are potential release sites into the lumen of the heart [21], suggesting hormonal release into the haemolymph and a dual role of PDFs as hormones and neuromodulators in velvet worms.

Despite their key phylogenetic position as members of Panarthropoda and Ecdysozoa (figure 1*a*), virtually nothing is known about the organization of the PDF/PDFR system in tardigrades. Three *pdf* homologues have been identified previously [21,39] in the genome and transcriptomes of the model eutardigrade [40,41] *Hypsibius exemplaris*, but their localization and functionalities are unknown. A cross-reactive antiserum, raised against the synthetic β-PDH peptide of the crustacean *Uca pugilator* [24] and commonly used for localizing PDF*-ir* neurons in various insects [4,5,8,10,11,22], crustaceans [24–26], onychophorans [21] and even molluscs [29], previously failed to detect the PDFs of tardigrades [21]. One *pdfr* orthologue was further identified *in silico* in the genome assemblies and predicted proteomes of two eutardigrade species, including *H. exemplaris* [39], but the existence of potential isoforms, their functionality and localization remain unexplored.

The present study thus sets out to close these gaps and to address the following questions: (i) are the three PDFs of *H. exemplaris* functional? (ii) Where are they localized and are any of them co-localized? (iii) How does the position of PDF-*ir* neurons and their projections relate to that in other protostomes, in particular insects and crustaceans? (iv) Are there differences in expression levels between the three *pdf* genes? (v) How do these compare to the expression levels of *pdfr* and its potential isoforms? (vi) Is PDFR localized in other than PDF-*ir* cells? This would suggest hormonal release of PDFs into the haemolymph, like in crustaceans [27], onychophorans [20], nematodes [28] and some insects [42,43]. Additional co-localization of PDFR with PDFs would imply autoreception, as reported from insects [44–47] and onychophorans [20]. Clarifying these questions will provide insights into the evolutionary changes in the organization and function of PDF/PDFR signalling across panarthropods and ecdysozoans.

## 2. Results

### 2.1. Orthology, composition and genomic location of *pdf* genes in eutardigrades

Genomic searches using three previously identified *pdf* sequences from the transcriptomes of *H. exemplaris* [21] as queries confirmed the existence of three *pdf* genes in this tardigrade species: *He-pdf-1*, *He-pdf-2* and *He-pdf-3*. To clarify orthology of identified homologues, we performed a phylogenetic analysis of *pdf* genes across eutardigrades. Genomic and transcriptomic screening, including reciprocal BLAST searches, revealed two to three *pdf* homologues in several species of Parachela (two in *Ramazzottius varieornatus* and *Mesobiotus philippinicus*, and three in *Richtersius coronifer*, *Paramacrobiotus richtersi* and *Paramacrobiotus metropolitanus*), whereas *Milnesium tardigradum* from the Apochela clade has two *pdf* homologues (figure 1*b*; electronic supplementary material, figure S1).

**Figure 2.**
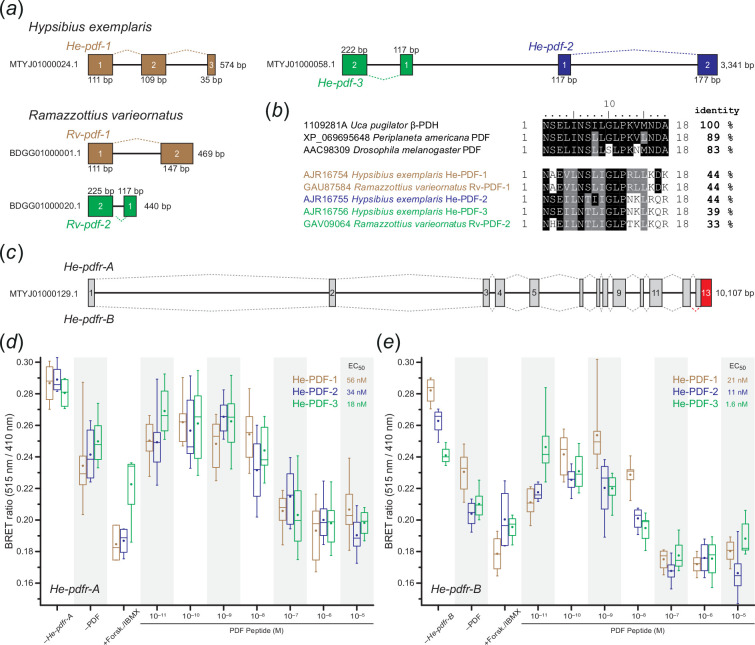
Genomic structure of *pdf* and *pdfr* genes of eutardigrades, and results of *in vitro* functional analyses of PDFs and PDFR isoforms in *H. exemplaris*. (*a*) Diagrams of genomic scaffolds illustrating the location and orientation of *pdf* genes in *H. exemplaris* and *Ramazzottius varieornatus*. Exons are depicted as coloured rectangles. Dashed lines indicate the position of introns. Note the proximity of *He-pdf-2* and *He-pdf-3* on a single scaffold (MTYJ01000058.1). (*b*) Comparison of amino acid sequences of mature tardigrade PDF peptides to selected arthropod PDF/PDHs. GenBank accession numbers are given for each peptide. Black and grey backgrounds indicate either identical or physicochemically similar amino acids to those of β-PDH from the crustacean *Uca pugilator*, respectively. Note the relatively low identities of tardigrade PDFs to β-PDH (33−44%). (*c*) Diagram of genomic structure of *pdfr* gene of *H. exemplaris*. Exons are depicted as rectangles. Dashed lines indicate the position of introns. Red rectangle (exon 13 of *He-pdfr-B*) indicates an alternative 3' splice junction (acceptor site) within the last exon of *He-pdfr* in *H. exemplaris*. (*d,e*) BRET ratio changes of HEK293T cells transfected with long (*He-pdfr-A*) and short variants (*He-pdfr-B*) of the PDF receptor from *H. exemplaris* after activation with synthetic PDFs (He-PDF-1, He-PDF-2 and He-PDF-3) in a dose-dependent manner. EC_50_ values are given for each peptide. Note decreasing BRET ratios (i.e. increasing intracellular cAMP levels) at higher concentrations of peptide indicating a stronger response to stimuli. Box plots of eight biological replicates (*n* = 8 for each dosage). Boxes correspond to interquartile ranges. The circle inside each box represents the arithmetic mean. Negative controls were performed without *pdfr* transfection (*n* = 4; *−He-pdfr-A; −He-pdfr-B*) and PDF stimuli (*n* = 8; −PDF). Positive controls were conducted with Forskolin/IBMX (at least *n* = 3; +Forsk./IBMX), which causes high intracellular concentrations of cAMP.

We further analysed the structure and arrangement of *pdf* genes in the assembled genomes of *H. exemplaris* and *Ramazzottius varieornatus*. The analysis revealed that *He-pdf-1* of *H. exemplaris* (574 bp) is located on the forward strand of scaffold0024 (GenBank accession number: MTYJ01000024.1), whereas *He-pdf-2* (1401 bp) and *He-pdf-3* (643 bp) are located on the forward and reverse strands of scaffold0058 (MTYJ01000058.1), respectively (figure 2*a*; electronic supplementary material, data S1). *He-pdf-1* consists of three exons (111 bp, 109 bp and 35 bp) separated by two introns (126 bp and 193 bp), while each of the two other *pdf* genes, *He-pdf-2* and *He-pdf-3*, comprise only two exons (*He-pdf-2*: 117 bp and 177 bp; *He-pdf-3*: 117 bp and 222 bp) separated by one intron (*He-pdf-2*: 1107 bp; *He-pdf-3*: 304 bp). In *Ramazzottius varieornatus*, the *Rv-pdf-1* gene (469 bp) is located on the forward strand of scaffold001 (BDGG01000001.1) and *Rv-pdf-2* (440 bp) on the reverse strand of scaffold020 (BDGG01000020.1), respectively. Each gene consists of two exons (*Rv-pdf-1*: 111 bp and 147 bp; *Rv-pdf-2*: 117 bp and 225 bp) separated by one intron (*Rv-pdf-1*: 211 bp; *Rv-pdf-2*: 98 bp) (figure 2*a*; electronic supplementary material, data S1).

### 2.2. One gene but two isoforms of pigment-dispersing factor receptor in *H. exemplaris*

After the initial BLAST screening, the complete coding sequence of the putative homologue of *pdf receptor* (*pdfr*) gene of *H. exemplaris*, *He-pdfr*, was obtained from each of the three publicly available transcriptome assemblies (GenBank accession numbers: GBZR01000000, GFGW01000000 and GJGU01000000). The existence of a single *pdfr* gene in *H. exemplaris* was confirmed by genomic searches (MTYJ00000000.1). The respective gene is 10 107 bp long and located on the reverse strand of scaffold0129 (MTYJ01000129.1). It consists of 13 exons and 12 introns (figure 2*c*; electronic supplementary material, data S1). Cloning and sequencing, however, revealed two transcribed isoforms, suggesting that at least two splice variants of PDFR are present in *H. exemplaris*, *He-pdfr-A* (1581 bp) and *He-pdfr-B* (1557 bp), the latter being eight amino acids (24 bp) shorter in the c-terminal region due to an alternative 3' splice junction (acceptor site) in the last exon of *He-pdfr* (figure 2*c*; electronic supplementary material, data S1). The orthologous gene *Rv-pdfr* of *Ramazzottius varieornatus* is considerably shorter (5341 bp) than *He-pdfr* of *H. exemplaris* and consists of 12 rather than 13 exons. Like *He-pdfr*, *Rv-pdfr* is located on the reverse strand of the respective scaffold (scaffold0005: BDGG01000005.1) and the *Rv-pdfr* transcript is of comparable length (1554 bp) to those of *He-pdfr-A* and *He-pdfr-B* (electronic supplementary material, data S1).

Using a similar strategy, we further identified two putative *pdfr* homologues in the transcriptomic and/or genomic databases from the eutardigrade *Paramacrobiotus metropolitanus* and one putative *pdfr* homologue in the heterotardigrades *Echiniscus testudo*, *Echiniscoides sigismundi* and *Batillipes* sp. To clarify the orthology of detected homologues, we performed a phylogenetic analysis of members of class B G protein-coupled receptors across bilaterians. In the resulting maximum likelihood tree, PDFRs occur as the sister clade to the corticotropin-releasing hormone receptors (figure 1*c*; electronic supplementary material, figure S2), thus confirming the identity of putative homologues from tardigrades, including both splice variants from *H. exemplaris*, as members of the *pdfr* clade.

### 2.3. Functionality of pigment-dispersing factors and pigment-dispersing factor receptor isoforms in *H. exemplaris*

To test the functionality of the two splice variants of PDFR and the three PDFs of *H. exemplaris*, we established bioluminescence resonance energy transfer (BRET) assays monitoring changes in the intracellular cAMP concentration upon stimulation with each PDF. Different dosages of synthetic He-PDF-1, He-PDF-2 and He-PDF-3 at final concentrations ranging from 10^−11^ M to 10^−5^ M (*n* = 8 for each dosage) induced PDFR/cAMP responses in the respective cell cultures (figure 2*d*,*e*; electronic supplementary material, data S1). Both receptor isoforms, He-PDFR-A and He-PDFR-B, were activated by each of the three peptides at low nanomolar concentrations (half maximal effective concentration (EC_50_) values: He-PDFR-A/He-PDF-1, 56 nM; He-PDFR-A/He-PDF-2, 34 nM; He-PDFR-A/He-PDF-3, 18 nM; He-PDFR-B/He-PDF-1, 21 nM; He-PDFR-B/He-PDF-2, 11 nM; He-PDFR-B/He-PDF-3, 1.6 nM) (figure 2*d*,*e*; electronic supplementary material, data S1).

### 2.4. Localization of pigment-dispersing factors and *pdf* mRNA in *H. exemplaris*

We used newly generated antibodies to localize the tardigrade PDFs. To test the specificity and potential cross-reactivity of these antibodies as well as the previously used β-PDH serum [21], we performed western blots with synthetic He-PDF-1, He-PDF-2 and He-PDF-3 (electronic supplementary material, figure S3*a*–*c*). For control experiments, the three specific PDF antibodies were applied reciprocally to the individual peptides in each blot. The results show that each of the three antibodies recognizes the corresponding peptide, indicated by a distinct band slightly below 4.6 kDa, while not binding to the non-corresponding PDFs (asterisks in electronic supplementary material, figure S3*a*–*c*). Like in the controls, the β-PDH serum does not show any staining (electronic supplementary material, figure S3*d*), suggesting that it does not cross-react with any of the three PDFs of *H. exemplaris*, although it was successfully used to detect PDFs in several protostomes including the cockroach *Periplaneta americana* [4] and the fruit fly *Drosophila melanogaster* [8]. The lack of cross-reactivity in the present study is consistent with the previous negative result [21] and is most likely due to the substantially altered PDF sequences in *H. exemplaris* (figure 2*b*).

**Figure 3.**
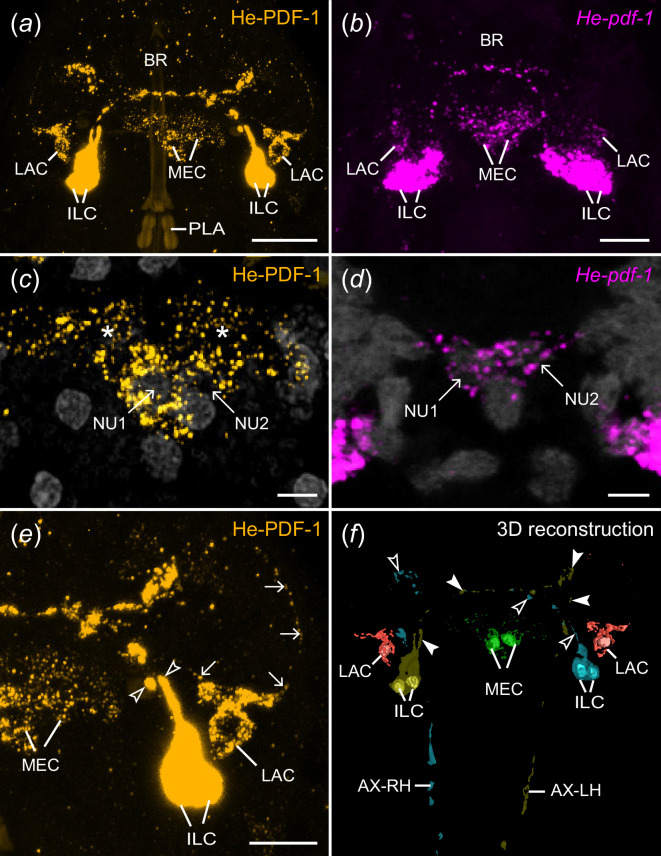
Localization of He-PDF-1 and *He-pdf-1* transcripts in the brain of the tardigrade *H. exemplaris*. Antibody (*a,c,e*) and mRNA labelling (*b,d*). 3D reconstruction (*f*) and projections of confocal substacks (*a–e*) in dorsal view. Anterior is up in all images. Pharyngeal placoids are autofluorescent in (*a*). DNA staining is illustrated in grey in (*c*) and (*d*). (*a*) Localization of the He-PDF1 peptide in inner lobe cells, lateral cells and median cells. (*b*) Localization of *He-pdf-1* mRNA. Note correspondence to immunolabelling in (*a*). (*c*) Detail of immunolabelled median cells with their nuclei (NU1 and NU2). Asterisks indicate varicosities of neurites labelled with He-PDF-1 antibody. (*d*) Detail of *He-pdf-1* mRNA expression in median cells. Note correspondence to immunolabelling in (*c*), except that varicosities have not been labelled. (*e*) Detail of right brain hemisphere from specimen in (*a*) showing individual axons of inner lobe cells (open arrowheads) and neurites of lateral cell (arrows). (*f*) 3D reconstruction of He-PDF-1 immunoreactivity illustrating number and position of somata of individual neurons with nuclei (coloured). Note immunoreactivity in two pairs of inner lobe cells (yellow and cyan), two median cells (green) and two lateral cells (red) within the brain. Arrowheads indicate varicosities of axonal projections of left (filled arrowheads) and right pairs of inner lobe cells (open arrowheads) that cross over to the other brain hemisphere. Abbreviations: AX-LH axonal projections from left brain hemisphere, AX-RH axonal projections from right brain hemisphere, BR brain, ILC inner lobe PDF-immunoreactive cells, LAC lateral PDF-immunoreactive cells, MEC median PDF-immunoreactive cells, NU1 nucleus of median cell 1, NU2 nucleus of median cell 2, PLA pharyngeal placoids. Scale bars: 10 µm (*a*), 5 µm (*b,e*) and 2 µm (*c,d*).

**Figure 4.**
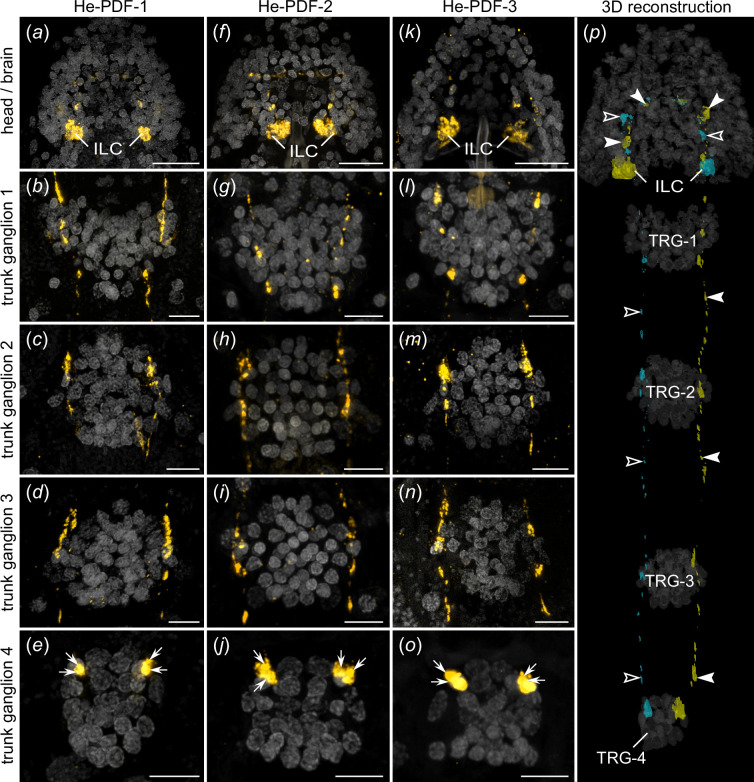
Localization of each of the three PDFs in the nervous system of *H. exemplaris*. Antibody labelling (orange in *a–o*), and 3D reconstruction (yellow and cyan in *p*). DNA staining is illustrated in grey. Projections of confocal substacks of the head region in dorsal view (*a,f,k*) and trunk ganglia in ventral view (*b–e,g–j,l–o*). Anterior is up in all images. (*a–o*) Note prominent expression of all three peptides in two pairs of somata in inner lobes of brain. Varicose axonal projections pass through first three trunk ganglia and terminate in anterior region of fourth trunk ganglion (arrows in *e,j,o*). (*p*) 3D reconstruction of left (yellow) and right pairs (cyan) of inner lobe cells and trajectories of their contralateral fibres (filled and open arrowheads) based on He-PDF-1 immunolabelling. Dorsal perspective. Note position of potential terminal buttons in anterior region of fourth trunk ganglion. Abbreviations: ILC inner lobe PDF-immunoreactive cells, TRG-1**–**TRG-4 first to fourth trunk ganglia. Scale bars: 10 µm (*a,f,k*) and 5 µm (*b–e,g–j,l–o*).

**Figure 5.**
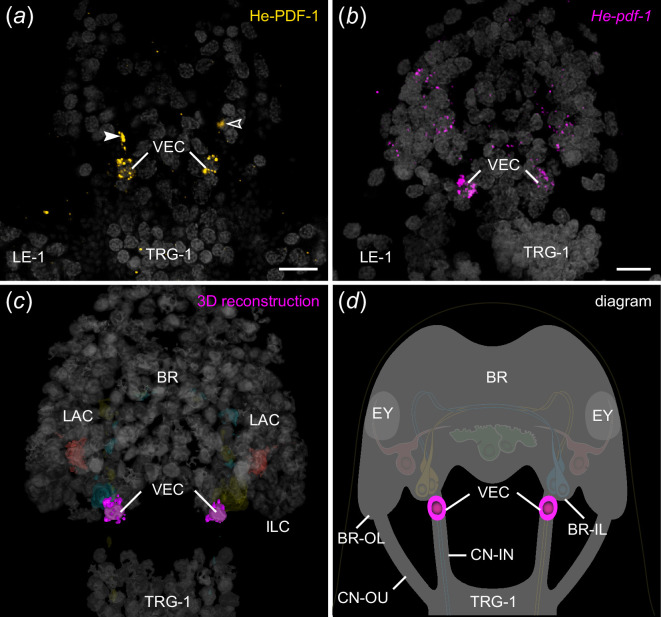
Localization of He-PDF-1 and *He-pdf-1* transcripts in the ventral extracerebral PDF-*ir* cells of *H. exemplaris*. Specimens are presented in ventral perspective. Anterior is up. Antibody labelling is illustrated in orange in (*a*), mRNA labelling in magenta in (*b*) and DNA staining in grey in (*a–c*). (*a,b*) Projections of confocal substacks. (*a*) Antibody labelling of He-PDF-1 peptide. Arrowheads indicate immunoreactive fibres of inner lobe cells in inner connectives. (*b*) Localization of *He-pdf-1* mRNA transcripts, which corresponds to distribution of He-PDF-1 peptide in (*a*). (*c*) 3D reconstruction of immunolabelled anterior body region. Note the position of somata of both ventral extracerebral PDF-*ir* cells outside the brain, anterior to first trunk ganglion. (*d*) Diagram of anterior body region illustrating position of ventral extracerebral PDF-*ir* cells that are widely separated from other PDF-*ir* cells across the *z*-axis. Abbreviations: BR brain, BR-IL inner lobe of brain, BR-OL outer lobe of brain, CN-IN inner connectives, CN-OU outer connectives, EY eyes, ILC inner lobe PDF-immunoreactive cells, LAC lateral PDF-immunoreactive cells, LE-1 first leg, TRG-1 first trunk ganglion, VEC ventral extracerebral PDF-immunoreactive cells. Scale bars: 5 µm (*b,c*).

Immunolocalization using specific antibodies revealed signal in several regions of the central nervous system and two extracerebral cells (figures 3–5). The most prominent signal occurs in the somata of two bilateral pairs of dorsal unipolar neurons (inner lobe cells) located in the posterior region of the inner lobes of the brain (figure 3*a*,*e*,*f*; electronic supplementary material, video S1). Each pair of these neurons sends out anteriorly their axons towards the contralateral region of the brain (figure 3*a*,*f*). The axons of each pair then fasciculate with those of their contralateral counterpart while passing through the anterior region of the central brain neuropil to the other brain hemisphere (figure 3*f*; electronic supplementary material, video S1). The two axonal pairs separate from each other and loop posteriorly descending towards the first trunk ganglion, to which they pass via the inner connectives (figure 3*f*). Each pair then passes through the contralateral side of each of the first three trunk ganglia, i.e. the two axons from the right brain hemisphere pass through the left side of each trunk ganglion and *vice versa* (figure 4*a*–*p*). The axons of the inner lobe cells terminate in two pairs of button-like structures in the antero-ventral region of the fourth trunk ganglion (arrows in figure 4*e*,*j* and *o*).

Besides one pair of inner lobe cells, each brain hemisphere of *H. exemplaris* exhibits one lateral and one median cell, totalling eight He-PDF1-*ir* neurons in the entire brain (figure 3*a*,*c*,*e*,*f*). The He-PDF-1*-ir* signal in the two median cells indicates anaxonic morphology, with numerous neurites in the anterior region of each cell but no apparent axon (figure 3*a*,*c*,*e*,*f*; electronic supplementary material, video S1). By contrast to the median cells, each lateral cell shows typical pseudounipolar structure, with a neurite arising from the soma and bifurcating into a median and a lateral process (arrows in figure 3*a*,*e*). We could not trace the entire path of the median neurite of the lateral cell, but it seems to enter the central neuropil, where most neurites of the median cells are located. The lateral neurite of the lateral cell shows an arcuate shape (arrows on the right of figure 3*e*). It terminates at the dorsal surface of the rhabdomeric cell of the eye, which is situated in the outer lobe of the brain (electronic supplementary material, figure S4). While the four inner lobe cells and their axons are immunoreactive to all three peptides (figure 4*a*–*o*; electronic supplementary material, figure S5), only He-PDF-1-*ir* signal (detectable at higher laser intensities) occurs in the two median and the two lateral cells, irrespective of the Zeitgeber time (electronic supplementary material, figure S6*a*–*c*).

In addition to the eight He-PDF-1-*ir* somata within the brain, there are He-PDF-1-*ir* somata outside the brain that belong to two individual cells. These ventral extracerebral cells are situated between the brain and the first trunk ganglion (figure 5*a*–*d*, electronic supplementary material, video S2). They are ventrally adjacent to the inner connectives of the central nervous system but do not show any PDF-*ir* fibres themselves. The PDF immunoreactivity in the inner connectives (arrowheads in figure 5*a*) solely derives from axons and varicosities of the inner lobe cells. Like the median and lateral cells within the brain, the ventral extracerebral cells show relatively weak He-PDF-1*-ir* signal and no anti-He-PDF-2 or anti-He-PDF-3 immunoreactivity.

**Figure 6.**
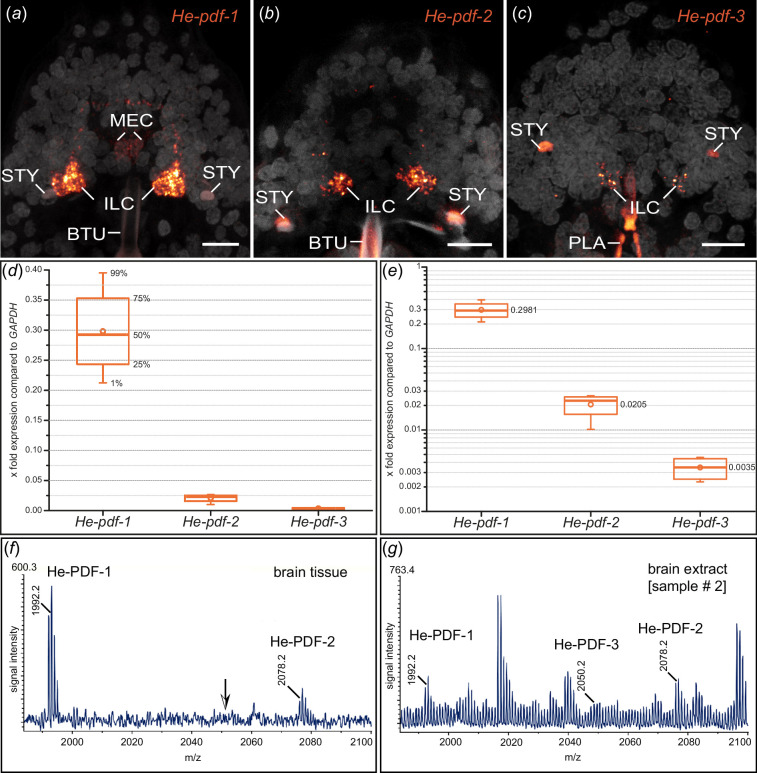
Differences in expression levels of the three *pdf* genes and MALDI-TOF mass spectra of the three PDFs in *H. exemplaris*. (*a–c*) Detection of *He-pdf-1*, *He-pdf-2* and *He-pdf-3* mRNA using same settings of confocal microscope. Brains in dorsal view. Anterior is up. DNA staining is shown in grey. Buccal tube, stylets and pharyngeal placoids are autofluorescent. Note that *He-pdf-1* shows highest and *He-pdf-3* lowest signal in inner lobe cells. (*d,e*) Relative expression levels of *He-pdf-1*, *He-pdf-2* and *He-pdf-3* based on comparative analysis of raw sequence reads from four independent RNA-seq experiments using short read mapping approach, with housekeeping gene *GAPDH* as reference. Boxes correspond to interquartile ranges. Circle inside each box represents arithmetic mean. (*d*) Linear chart. (*e*) Logarithmic chart using same dataset as in (*d*). (*f,g*) Representative MALDI-TOF mass spectra obtained from single dissected brain by direct tissue profiling (*f*) and extract of 100 brains (*g*) from sample no. 2 (see §4; electronic supplementary material, figure S10). Ion signals are marked and represent single charged peptides [M+H]^+^. Note that using direct tissue profiling only He-PDF-1 and He-PDF-2 could be detected, whereas ion masses corresponding to all three predicted He-PDFs were obtained from brain extracts. Abbreviations: BTU buccal tube, ILC inner lobe PDF-immunoreactive cells, MEC median PDF-immunoreactive cells, PLA pharyngeal placoids, STY stylet. Scale bars: 5 µm (*a–c*).

To further substantiate the results of immunolabelling, we performed various sets of *in situ* hybridization experiments. The mRNA labelling essentially revealed the same results, except that neuronal projections are labelled weaker using this technique as compared with the immunolabelling. All three complementary probes to the *He-pdf-1*, *He-pdf-2* and *He-pdf-3* transcripts are co-localized in the inner lobe cells, whereas the median cells, the lateral cells and the ventral extracerebral cells exhibit only *He-pdf-1* signal (figures 3*b*,*d* and 5*b*; electronic supplementary material, videos S3 and S4). This finding is consistent in single and double *in situ* hybridization experiments (electronic supplementary material, figures S7 and S8) as well as assays using a combination of the *He-pdf-1* probe with the He-PDF-1 antibody (electronic supplementary material, figure S9). Like the immunostaining experiments, mRNA labelling in specimens at different Zeitgeber times revealed the most prominent *He-pdf-1* signal in the somata of the inner lobe cells, whereas the median and lateral cells show relatively weak staining (electronic supplementary material, figure S6*d*–*f*). *He-pdf-1* signal is consistently stronger in somata of the inner lobe cells (*n* = 15), whereas *He-pdf-2* (*n* = 5), and especially *He-pdf-3* (*n* = 5) show relatively weaker signals, even at higher concentrations of the respective probes and laser intensities used (figure 6*a*–*c*; electronic supplementary material, table S1). The same holds true for the results of double labelling with each two of the three probes (electronic supplementary material, figure S8). This noticeable difference in relative signal intensities corresponds to the numbers of short sequencer reads we quantified from the available transcriptomes using a read mapping approach, which revealed nearly a 15 times lower expression of *He-pdf-2* and an 86 times lower expression of *He-pdf-3* compared with *He-pdf-1* (figure 6*d*,*e*; electronic supplementary material, data S2).

### 2.5. Identification of pigment-dispersing factors in brain samples from *H. exemplaris* by mass spectrometry

To confirm the presence of putative PDFs in the brain, we first analysed samples of entire brains using direct tissue profiling by matrix-assisted laser desorption/ionization (MALDI) time of flight (TOF) mass spectrometry (MS). This revealed a typical spectrum in a mass range at m/z 1800−2100 Da (figure 6*f*,*g*). The most abundant ion signal originated from He-PDF-1, followed by He-PDF-2, whereas the predicted ion mass of He-PDF-3 could not be detected (arrow in figure 6*f*). Further fragmentation experiments using MALDI-TOF/TOF MS to determine the amino acid sequences were unsuccessful due to the low concentration of peptide in the sample spot. We then prepared brain extracts to increase the concentration of peptides in the sample sets. The resulting MALDI-TOF mass spectrum revealed all three predicted PDFs, where He-PDF-1 shows the most abundant ion signal, followed by He-PDF-2 and He-PDF-3 (figure 6*g*). This corresponds well to the results of mRNA detection (figures 3*e* and 6*a*–*c*) as well as quantitative analyses of expression levels (figure 6*d*,*e*). For chemical identification of transcriptome-predicted PDFs, we analysed brain extracts by ESI-Q Exactive Orbitrap MS, followed by an evaluation of peptide fragmentation using PEAKS 10.5 software package. This resulted in a confirmation of amino acid sequences of all three PDFs (electronic supplementary material, figure S10).

### 2.6. Quantification and localization of pigment-dispersing factors receptor in *H. exemplaris*

**Figure 7.**
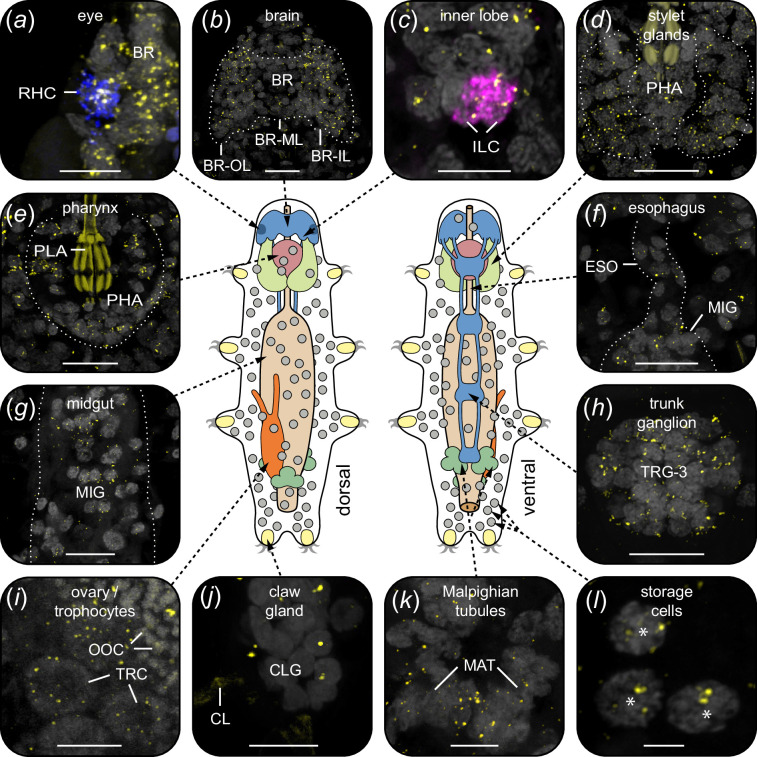
Localization of PDF receptor mRNA in various tissues and cells of *H. exemplaris*. Labelling using *He-pdfr* probes is illustrated in yellow in (*a–l*) (see electronic supplementary material, figure S15 for positive controls). Projections of confocal substacks (*a–l*) and diagrams of tardigrade anatomy in dorsal and ventral views (centre). Dotted white lines indicate contours of organs and tissues. Anterior is up in all images. Grey colour shows DNA staining in (*a–h)*, (*j*) and (*k*), and autofluorescence in (*i*) and (*l*). Pharyngeal placoids are autofluorescent in (*d*) and (*e*), and claws in (*j*). (*a*) Expression of *He-pdfr* in the rhabdomeric cell of the eye labelled with *r-opsin-v* mRNA probe (blue). (*b*) Expression of *He-pdfr* in the brain. (*c*) Expression of *He-pdfr* in inner lobe cells double labelled with *He-pdf-1* probe (magenta). (*d–l*) Expression of *He-pdfr* in various other tissues and cells. Asterisks indicate storage cells that occur within body cavity. Abbreviations: BR brain, BR-IL inner lobe of brain, BR-ML median lobe of brain, BR-OL outer lobe of brain, CLG claw gland, CL claw (visualized by cuticular autofluorescence), ESO esophagus, ILC inner lobe PDF-immunoreactive cells, MAT Malpighian tubules, MIG midgut, OOC autofluorescent yolk granules of an oocyte, PHA pharynx, PLA pharyngeal placoids, RHC rhabdomeric cell, TRG-3 third trunk ganglion, TRC trophocytes. Scale bars: 10 µm (*b,d–h*), 5 µm (*a,c,i–k*) and 2 µm (*l*).

Quantitative analysis of four transcriptomes of *H. exemplaris* revealed similar expression levels of both splice variants of the PDFR, *He-pdfr-A* and *He-pdfr-B*, each of which is approximately 14 times lower than that of *He-pdf-1* (electronic supplementary material, figure S11 and data S2). To localize cells that express *pdfr*, we conducted hybridization chain reaction–fluorescence *in situ* hybridization (HCR-FISH) using DNA split initiator *He-pdfr* probes. Due to the small difference of 24 nucleotides, we could not distinguish between the two isoforms of the receptor. Our data show a widespread expression of *pdfr* in all major organs and tissues of *H. exemplaris*, including the eye, the outer, inner and median lobes of the brain, the four trunk ganglia, the somatic muscles, the digestive tract (pharynx, oesophagus and midgut), the trophocytes of the ovary, the Malpighian tubules, the stylet glands and the claw glands (figure 7*a*–*k*; electronic supplementary material, figure S12). We further localized *He-pdfr* mRNA in storage cells that are abundant in the body cavity (figure 7*l*). Double HCR-FISH revealed co-expression of *He-pdfr* with visual *r-opsin* (*He-r-opsin-v*) in the rhabdomeric cell of the eye (figure 7*a*; electronic supplementary material, figure S13). The same technique exhibited *He-pdfr* mRNA in somata of all PDF-*ir* neurons within the brain, including the inner lobe cells, the lateral cells and the median cells (figure 7*c*; electronic supplementary material, figure S14). The results of positive control experiments using two sets of redundant probes are consistent and confirm the specificity of our *He-pdfr* probes (electronic supplementary material, figure S15).

## 3. Discussion

### 3.1. Scenario on the evolution and duplication of *pdf* genes in tardigrades

Comparison with previous findings [21] allows us to expand the scenario on the evolution of *pdf* genes (figure 1*a*). While only one *pdf* gene was most likely present in the last common ancestor of protostomes, a duplication might have led to two genes in the ecdysozoan lineage, *pdf-I* and *pdf-II*, whose homologues are still present in extant priapulids, nematodes and onychophorans [20,21]. Subsequently, *pdf-II* was lost whereas *pdf-I* has been retained in tardigrades and arthropods, followed by independent duplications in each of these taxa [21]. While *pdf* genes have not been characterized in heterotardigrades, the last common ancestor of eutardigrades likely possessed two *pdf-I* homologues, one of which was duplicated either once again in the parachelan lineage, followed by subsequent losses in some species (*Ramazzottius varieornatus* and *Mesobiotus philippinicus*), or multiple times independently in separate lineages, including those containing *Richtersius coronifer*, *H. exemplaris* and *Paramacrobiotus* species.

Although a conclusive scenario on the evolution of *pdf* genes is hard to depict due to the still unresolved internal phylogeny of Parachela [48], a single duplication of the parachelan *pdf-2/3* precursor, followed by subsequent species- or lineage-specific losses of either *pdf-2* or *pdf-3*, is in line with the number of identified *pdf* genes in different eutardigrade species. This scenario receives additional support from the topology of our maximum likelihood tree, in which parachelan PDFs occur in two distinct clades (PDF-1 and PDF-2/3). Despite the clear indication of two *pdf-I* paralogues (*pdf-1*, and *pdf-2/3* precursor) in the last common ancestor of eutardigrades, we caution that this scenario is still based on a limited number of sequenced tardigrade genomes and relatively short peptide sequences, consisting only of 18 amino acids. Hence, our hypothesis on the evolution and duplication of *pdf* genes in tardigrades will have to be further tested once additional genomes, especially from heterotardigrades and apochelan eutardigrades, have become available.

### 3.2. Homology of protocerebral pigment-dispersing factor-immunoreactive neurons and the origin of ventral extracerebral pigment-dispersing factor-immunoreactive cells in *H. exemplaris*

**Figure 8.**
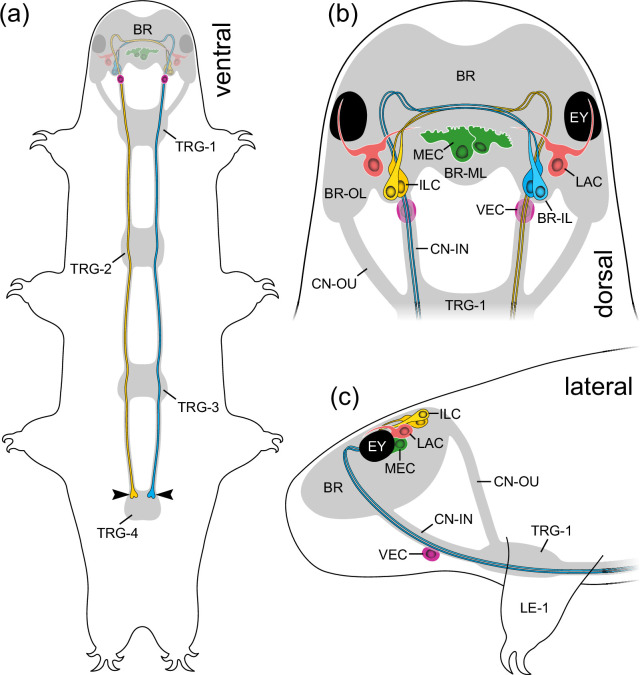
Summary diagrams of localization of PDFs in the head of *H. exemplaris*. Brain, trunk ganglia and connectives are indicated in grey. (a) Overview illustrating the position of PDF-*ir* neurons (coloured), ventral extracerebral PDF-*ir* cells and trajectories of inner lobe cells (yellow and blue). Arrowheads point to button-like terminals on the antero-ventral surface of the fourth trunk ganglion. (b) Diagram illustrating the number, position, morphology and connectivity of PDF-*ir* cells in the head in dorsal view. Note eight cerebral neurons exhibiting different morphologies and two ventral extracerebral cells associated with inner connectives. Note also that lateral PDF-*ir* neurons send their projections to the eyes and central part of the brain. (c) Same as in (b), but in lateral view. Abbreviations: BR brain, BR-IL inner lobe of brain, BR-ML median lobe of brain, BR-OL outer lobe of brain, CN-IN inner connectives, CN-OU outer connectives, EY eye, ILC inner lobe PDF-immunoreactive cells, LAC lateral PDF-immunoreactive cells, LE1 first leg, MEC median PDF-immunoreactive cells, TRG-1**–**TRG−4 first to fourth trunk ganglia, VEC ventral extracerebral PDF-immunoreactive cells.

We have demonstrated that the three *pdf* homologues of *H. exemplaris*, which are derivatives of *pdf-I* of the last common ancestor of Ecdysozoa [21] (figure 1*a*), exhibit different expression patterns. While *He-pdf-1*, *He-pdf-2* and *He-pdf-3* are co-expressed in the two pairs of inner lobe cells, only *He-pdf-1* mRNA is found in the median and lateral cells as well as the ventral extracerebral PDF*-ir* cells (figure 8a–c). The results of mRNA labelling are entirely consistent with those based on the immunolocalization of PDFs, demonstrating that eight out of 10 PDF*-ir* somata of *H. exemplaris* are located in the brain. Given that the tardigrade brain most likely consists of a single segmental region homologous with the protocerebrum of other panarthropods [49], the existence of protocerebral He-PDF-*ir* neurons corresponds to that in onychophorans [20,21] and most arthropods [26,32,42,43,50]. This suggests that several bilateral PDF-*ir* somata were present in the protocerebrum of the last common ancestor of Panarthropoda. Provided that the median protocerebral PDF-*ir* cells are homologous across panarthropods, their absence in *Drosophila melanogaster*, *Rhyparobia madeira*, *Apis mellifera*, *Acyrthosiphon pisum* [5,8,13,17] and various other insects [4,22] most likely resulted from convergent losses in the corresponding taxa.

The two ventral extracerebral PDF-*ir* cells associated with the inner connectives of *H. exemplaris* (figure 8b,c) are most likely a derived feature of tardigrades or a tardigrade subclade, as such extraganglionic cells situated between the brain and the ventral nervous system have not been reported from any other animal group. These cells might correspond to a pair of ‘anteroventral cells’ that arise early in the embryo of *H. exemplaris* (formerly referred to as *H. dujardini* [40]) and become connected to the first trunk ganglion as well as the circumbuccal nerve ring [51]. These embryonic cells give rise to neurites extending towards the body surface later in development, suggesting they are sensory neurons. Our labelling did not reveal any PDF-*ir* fibres associated with the ventral extracerebral cells, which might be due to the relatively weak staining of these cells. Thus, the identity, detailed morphology and potential function of the ventral extracerebral PDF-*ir* cells remain to be clarified.

### 3.3. Pigment-dispersing factors of tardigrades are neurohormones and neuromodulators that control various body functions

The results of our *in vitro* assays using BRET in transfected human cells indicate that all three PDFs of *H. exemplaris* and both splice variants of their receptor are functional and that G protein-coupled receptor signalling (in this case PDF/PDFR signalling) based on cAMP regulation is present in tardigrades. Moreover, the respective EC_50_ values estimated for both splice variants of *He-pdfr* are comparable with PDF/PDFR activation at low nanomolar concentrations reported from other animals (1.6−56 nM in *H. exemplaris*, 12.8 nM in *Bombyx mori* [52], approx. 25 nM in *Drosophila melanogaster* [46], 20−40 nM in *Carcinus maenas* [27]). However, our expression level analyses revealed substantial differences in the abundance of the three peptides in *H. exemplaris*, with *He-pdf-2* showing approximately 15 times and *He-pdf-3* 86 times lower values than *He-pdf-1*. This, together with different EC_50_ values among the three peptides, indicates a higher potency of *He-pdf-3* (lowest EC_50_, lowest abundance) compared with *He-pdf-2* (medium EC_50_, medium abundance) and *He-pdf-1* (highest EC_50_, highest abundance). The functional role of these differences between the three peptides as well as their different localization in the head (figure 8a–c) is unclear. However, they might still be subject to distinct regulatory mechanisms *in vivo*, i.e. in the tardigrade body, which we did not investigate.

Our data further revealed that PDFR of *H. exemplaris* is expressed in all major organ systems and tissues of the body, including storage cells. Co-localization of PDFR with all three PDFs in somata of the inner lobe cells and with He-PDF-1 in the median and lateral cells suggests an autoregulatory signalling loop in tardigrades. This might resemble the PDF/PDFR autoreception in the insect clock [44,53,54], although PDF and PDFR are co-localized only in a subset of PDF-*ir* clock neurons in insects [45,46]. Analysing the expression of core and associated clock genes in *H. exemplaris* may help to clarify whether PDF-*ir* neurons are involved in the circadian system of tardigrades.

The spatial separation of PDF-producing neurons and *pdfr-*expressing cells suggests that the PDFs of tardigrades act as neurohormones that might be released into the body cavity for targeting other cells. We have indeed detected two pairs of prominent button-like structures on the antero-ventral surface of the fourth trunk ganglion (arrowheads in figure 8a) that represent the axonal terminals of the inner lobe cells, the somata of which are located in the brain. These paired, unipolar neurons project axons to the contralateral brain hemisphere, which then follow each inner connective and pass through the first three trunk ganglia to finally terminate in the fourth trunk ganglion (figure 8a–c). We speculate that these button-like structures are potential release sites of all three peptides, as the inner lobe cells are the only neurons that produce all three of them at levels detected in our assays and via our antibodies. Thus, the inner lobe cells might play a neuroendocrine role in *H. exemplaris*. Their contralaterally projecting axons suggest that these cells might play a role in coupling both brain hemispheres, like the PDF-*ir* neurons in the circadian clock of various insects [4–8,31]. Homologues of corpora cardiaca or corpora allata have not been identified in tardigrades but the terminals of the inner lobe cells might have a similar neurosecretory function [42,43]. We caution, however, that the involvement of PDFs in the function of the circadian clock of tardigrades remains to be demonstrated.

Localization of *pdfr*-expressing target cells in all major tissues and organs of *H. exemplaris* [55] further suggests that PDFs might control or synchronize various functions in tardigrades, such as detection of light, neural processing, locomotion, feeding, digestion, osmoregulation, growth, development, oogenesis/reproduction, and formation of stylets and claws—a process related to moulting [56]. The role of PDF/PDFR signalling in storage cells is unclear, but since these cells have been mainly associated with the production, storage and transport of lipids, polysaccharides and proteins [57,58], PDFs might additionally regulate the storage and distribution of nutrients in the tardigrade body. This wide variety of functions parallels the reported multiple roles of PDFs in nematodes, ranging from the control of locomotion and mate searching to mechano- and chemosensation, including the sensation of oxygen [28,36,37]. A recent study further revealed that PDFR is expressed in multiple organs of the fruit fly *Drosophila melanogaster* [59], suggesting hormonal control and multiple functions of the sole PDF, which like the three PDFs of *H. exemplaris* has originated from *pdf-I* of the ecdysozoan ancestor [21]. Similar observations, though without functional analyses, have been made in onychophorans [20]. These findings, together with our present observations, suggest a neuroendocrine role and multifunctionality of PDFs in the last common ancestors of Panarthropoda and Ecdysozoa. Focusing on multiple rather than just clock-related roles of PDFs in distantly related species would help to clarify the variety of functions and evolution of PDF/PDFR systems across protostomes.

## 4. Methods

### 4.1. Rearing and collection of specimens

Specimens of the tardigrade species *Hypsibius exemplaris* [40] (Eutardigrada, Hypsibiidae; strain Z151, Sciento, UK) were maintained under a 12/12 h light/dark cycle regime and fed with algae (*Chlorococcum* sp.) as described previously [60]. Approximately 50 adult specimens were collected with a glass pipette under a stereomicroscope for each localization experiment (immunolabelling and *in situ* hybridization), whereas several hundred specimens were extracted using a gauze (mesh size: 50 µm) for RNA extraction and purification.

### 4.2. Transcriptomic, genomic and phylogenetic analyses

The coding sequences of three *pdf* homologues (*He-pdf-1*: KP266565; *He-pdf-2*: KP266566; and *He-pdf-3*: KP266567) of *H. exemplaris* were obtained from the publicly available NCBI (National Center for Biotechnology Information) repository [21]. To clarify the orthology and evolutionary history of *pdf* genes across eutardigrades, we searched for putative *pdf* orthologues in the assembled transcriptomes from several tardigrade species using either tBLASTn or BLASTp v. 2.12.0+ [61] and the three *pdf* homologues of *H. exemplaris* as queries (electronic supplementary material, table S2). We further applied a previous methodology [21] to obtain the best maximum likelihood tree using RAxML v. 8.2.12 [62] and raxmlGUI v. 2.0.10 [63], but expanded the dataset by including *pdf* sequences from additional species of tardigrades and other panarthropods and aligned all sequences using the MAFFT online version [64] (G-INS-i option) (electronic supplementary material, table S2). After inferring the best tree from 100 independent runs under empirical LG+G substitution model (according to the model test implemented in raxmlGUI), bootstrap support values were estimated from 100 pseudoreplicates of the original alignment. The resulting tree was visualized using iTol v. 6 [65] and edited with Illustrator CS5.1 (Adobe Inc., San Jose, CA, USA).

Available transcriptome assemblies of *H. exemplaris* (GenBank accession numbers: GBZR01000000, GFGW01000000 and GJGU01000000) were used to search for putative *pdfr* transcripts. Initially, tBLASTn v. 2.12.0+ searches [61] were conducted against all three assemblies using sequences from the onychophoran *Euperipatoides rowelli* (GenBank accession number: MT080366.1) and the fruit fly *D. melanogaster* (NP_570007.2) as query sequences. Afterwards, the putative *pdfr* transcript of *H. exemplaris* was used as query for BLAST searches in genomic scaffolds of *Ramazzottius varieornatus* (BDGG00000000.1) and *Paramacrobiotus metropolitanus* (BHEN00000000.1) to obtain the putative *pdfr* genes of the respective species. For heterotardigrades, the transcriptome assemblies of *Echiniscus testudo* and *Echiniscoides sigismundi* were obtained from publicly available databases and an unpublished genome assembly of *Batillipes* sp. (electronic supplementary material, table S2). Thereafter, tBLASTn v. 2.12.0+ searches [61] were performed using the putative *pdfr* sequences from *H. exemplaris* and the previously reported *pdfr* sequence from *Euperipatoides rowelli* [20] as bait sequences. The blast search yielded 21 hits for *Echiniscus testudo*, 20 hits for *Echiniscoides sigismundi* and eight hits for *Batillipes* sp. Subsequently, reciprocal BLAST searches with the resulting hits against the nucleotide database (nr/nt) of NCBI were performed to identify putative *pdfr* genes from heterotardigrades.

The transmembrane domains of putative *pdfr* sequences of *H. exemplaris*, *Paramacrobiotus metropolitanus*, *Echiniscus testudo*, *Echiniscoides sigismundi* and *Batillipes* sp. were predicted using the SMART online database [66] and included in a previous dataset of approximately 1000 class B G protein-coupled receptors obtained from a cluster analysis of approximately 18 000 bilaterian G protein-coupled receptors, including PDFRs [20]. After aligning the dataset using the MAFFT online version [64] (G-INS-i option) a combined maximum likelihood analysis of 10 independent inferences and 100 thorough bootstrap pseudoreplicates was conducted under a dataset specific GTR+G model using RAxML v. 8.2.12 [62] and raxmlGUI v. 2.0.10 [63]. The resulting tree was visualized using iTol v. 6 [65] and edited with Illustrator CS5.1 (Adobe Inc.). To analyse the genomic structure and to clarify whether there are any additional copies of the studied *pdf* and *pdfr* genes, BLASTn searches against the whole genomes of *H. exemplaris* (MTYJ00000000.1) and *Ramazzottius varieornatus* (BDGG00000000.1) were performed and intron/exons identified in the obtained genomic scaffolds by direct comparison of transcriptomic and genomic sequences of the corresponding genes (electronic supplementary material, data S1).

### 4.3. Amplification and cloning of *pdf* and *pdfr* fragments

Total RNA was isolated from several hundred specimens of *H. exemplaris* with TRIzol^®^ reagent (Thermo Fisher Scientific, Waltham, MA, USA) and resulting RNA was purified with RNeasy^®^ MinElute^®^ Cleanup Kit (Qiagen, Hilden, Germany) according to manufacturer’s protocols. The concentration of the purified RNA was measured with a Qubit 3.0 Fluorometer (Life Technologies, Thermo Fisher Scientific). The first-strand cDNA synthesis from purified RNA was performed using random hexamer primers (3 μg ml^−1^) and SuperScript IV reverse transcriptase (Thermo Fisher Scientific) according to the manufacturer’s instruction. Subsequently, full-length coding sequences of the target genes were amplified with gene-specific forward and reverse primers from previously obtained first strand cDNA (electronic supplementary material, table S3). The amplicons of *He-pdf-1, He-pdf-2* and *He-pdf-3* were ligated into pJET1.2 vector (Thermo Fisher Scientific) as per manufacturer’s specification and transferred to competent *E. coli* TOP10 cells for overnight growth in lysogeny broth plates at 37°C. Several colonies were picked to perform colony PCR and selected clones were verified by Sanger sequencing (Eurofins Genomics GmbH, Ebersberg, Germany). The amplification by designing gene-specific primers, cloning, and sequencing of the *He-pdfr* fragments were conducted using the same approach to obtain full-length coding sequences (electronic supplementary material, table S3).

### 4.4. Quantitative analysis of *pdf* and *pdfr* transcripts

The abundance of the identified *He-pdf-1*, *He-pdf-2*, *He-pdf-3* and *He-pdfr-A/B* transcripts was estimated using segemehl 0.3.4 [67] as described previously [68]. In brief, the raw sequence reads from four independent RNA-seq experiments of *H. exemplaris* (Sequence Read Archive run accession numbers: SRR14868527, SRR5218239, SRR5218240 and SRR5218241) were mapped back on the corresponding sequences allowing for a maximum of 5% mismatch of nucleotides per read (electronic supplementary material, data S2). Subsequently, the relative abundance (matched nucleotides per position and giga base pair) of genes was normalized using the relative abundance of the housekeeping gene *glyceraldehyde-3-phosphate dehydrogenase* (*GAPDH*) as a reference (100%). We have chosen this gene, as it shows the lowest variation among the tested putative reference genes (*GAPDH* and 29 ribosomal protein genes) in the studied transcriptomes, although the relative expressions of *He-pdfs* do not differ much among the six most stable reference genes (*He-GAPDH*, *He-RPL9*, *He-RPL18A*, *He-RPL13A*, *He-RPL15*, *He-RPS23*; electronic supplementary material, data S2). The results were visualized as box plots at linear and logarithmic scales.

### 4.5. Matrix-assisted laser desorption ionization-time of flight mass spectrometry

Tardigrades were anaesthetized on ice. Each specimen was then transferred to a droplet of ice-cold physiological insect saline (128 mM NaCl, 2.7 mM KCl, 2 mM CaCl_2_, 1.2 mM NaHCO_3_, pH 7.25) and placed in a dissection dish filled with Sylgard 184 Silicone Elastomer (Dow Corning, USA). Close to its posterior end, the tardigrade was fixed using an ultra-fine pin (Sphinx V2A, 0.1 × 12.0 mm, Czech Republic). Subsequently, the head was opened laterally using an ultra-fine scissor (Fine Science Tools, no. 15000-08, Heidelberg, Germany) under a stereomicroscope (Zeiss Stemi 305; Carl Zeiss Microscopy GmbH, Jena, Germany). For direct tissue profiling experiments (*n* = 15), brains were fixed using an ultra-fine forceps (Fine Science Tool, No. 11254-20) and cut out carefully using the ultra-fine scissor. Then, the brain was divided into smaller portions using the ultra-fine scissor. Thereafter, each tissue sample was transferred into a drop of water placed on a sample plate for MALDI-TOF MS analysis using a glass capillary fitted to a tube with a mouthpiece as described in detail for small tissue samples [69] and direct single cell analysis in insects [70]. Immediately after transfer, water was removed from around the sample and the tissue was allowed to air-dry prior to the application of the matrix.

For brain extraction (*n* = 2), two samples consisting of 100 brains each were prepared. Brains were dissected as described above and transferred with a glass capillary fitted to a tube with a mouthpiece into 30 μl extraction solution on ice. Sample no. 1 contained 50% methanol, 49% H_2_O and 1% formic acid (FA), whereas sample no. 2 contained 90% ethanol, 9% H_2_O and 1% acetic acid. Tissue samples were homogenized using an ultrasonic bath (Transonic 660/H, Elma Schmidbauer GmbH, Hechingen, Germany) for 90 min on ice. Afterwards, the samples were centrifuged for 15 min at 13 000 rpm at 4°C. The supernatants were separated and then evaporated in a vacuum concentrator to remove organic solvent. Extracts were stored at −20°C until use. For matrix application, we followed a previous protocol [71], except that we applied only 10 mg ml^−1^ 2,5-dihydroxybenzoic acid (DHB; Sigma-Aldrich, Steinheim, Germany) dissolved in 20% acetonitrile, 1% FA and 79% H_2_O (Fluka) as matrix. For direct tissue profiling, dried tissue samples were covered with 0.1 µl 2,5-dihydroxybenzoic acid solution applied with a 0.1−2.5 µl pipette (Eppendorf, Hamburg, Germany). For extract analysis, 0.1 µl concentrated supernatant was mixed with 0.1 µl DHB solution and again applied with a 0.1−2.5 µl Eppendorf pipette. Subsequently, sample spots were dried with a commercially available hair dryer to form homogeneous crystals.

Mass spectra were acquired manually using an ultrafleXtreme TOF/TOF mass spectrometer (Bruker Daltonik GmbH, Bremen, Germany) in reflector positive ion mode in a mass range of 900−3000 Da. For calibration, the following mixture of synthetic peptides was used: (i) short neuropeptide F receptor of *Drosophila melanogaster*, (Drm)-sNPF-1 [4–11]; (ii) periviscerokinin-1 of *Locusta migratoria* (Lom)-PVK-1; (iii) FMRFamide-12 of *Periplaneta americana*, (Pea)-FMRFa-12; (iv) allatotropin of *Manduca sexta* (Mas)-AT; (v) IPNamide of *Drosophila melanogaster*, (Drm)-IPNa; (vi) SKN of *Periplaneta americana*, Pea-SKN; and (vii) human glucagon. Laser fluency was adjusted to provide an optimal signal-to-noise ratio. The obtained data were processed using FlexAnalysis v. 3.4 software package (Bruker Daltonik GmbH).

#### 4.5.1. Quadrupole orbitrap mass spectrometry coupled to nanoflow high-performance liquid chromatography

For fragmentation analysis and sequence evaluation, brain extracts were desalted using self-packed Stage Tip C18 (IVA Analysentechnik e.K., Meerbusch, Germany) spin columns [72] before injecting the samples into the nanoLC system. For analysis, peptides were separated on an EASY nanoLC 1000 UPLC system (Thermo Fisher Scientific) using 50 cm RPC18 columns (fused Silica tube with ID 50 ± 3 μm, OD 150 ± 6 μm, Reprosil 1.9 μm, pore diameter 60 Å, Dr Maisch, Ammerbuch-Entringen, Germany) and a binary buffer system (A: 0.1% FA, B: 80% ACN, 0.1% FA) as described for *Cataglyphis nodus* samples [71]. Running conditions were as follows: linear gradient from 2% to 62% B in 110 min, 62% to 75% B in 30 min, and final washing from 75% to 95% B in 6 min (45°C, flow rate 250 nl min^−1^). Finally, the gradients were re-equilibrated for 4 min at 5% B. High-performance liquid chromatography (HPLC) was coupled to a Q-Exactive Plus (Thermo Scientific, Bremen, Germany) mass spectrometer. MS data were acquired in a top-10 data-dependent method dynamically choosing the most abundant peptide ions from the respective survey scans in a mass range of 300−3000 m/z for higher-energy collisional dissociation (HCD) fragmentation. Full MS acquisitions ran with a resolution of 70 000, automatic gain control target (AGC target) at 3e6, and maximum injection time at 80 ms. HCD spectra were measured with a resolution of 35 000, AGC target at 3e6, maximum injection time at 240 ms, 28 eV normalized collision energy, and dynamic exclusion set at 25 s. The instrument was run in peptide recognition mode (i.e. from two to eight charges), singly charged and unassigned precursor ions were excluded. Raw data were analysed with PEAKS Studio 10.5 (BSI, ON, Canada). Neuropeptides were searched against an internal database comprising neuropeptide precursor sequences from tardigrades with parent mass error tolerance of 0.2 Da and fragment mass error tolerance of 0.2 Da. Setting enzymes: none was selected because samples were not digested. The false discovery rate (FDR) was enabled by a decoy database search as implemented in PEAKS 10.5. The following posttranslational modifications (PTM) were selected: C-terminal amidation as fixed PTM and oxidation at methionine, phosphorylation, sulfation as variable PTMs. In each run a maximum of three variable PTMs per peptide were allowed. Fragment spectra with a peptide score (−10 lgP) equivalent to a *p*-value of approximately 1 %, were manually reviewed.

### 4.6. Bioluminescence resonance energy transfer assays

We tested the functionality and potential differences in interaction of the He-PDF-1, He-PDF-2 and He-PDF-3 peptides with both splice variants of their receptor, He-PDFR-A and He-PDFR-B. For this purpose, we established BRET assays using an Epac-based sensor (Epac-L) to examine cAMP responses in transfected human cells (HEK293T) as described previously [20] for the onychophoran *Euperipatoides rowelli*. A POLARstar Omega microplate reader (BMG Labtech, Cary, NC, USA) was used to measure the resulting raw luminescence responses after stimulating the transfected cells with different concentrations (10^−11^−10^−5^ M) of synthetic PDFs [20] (Biomatik Corp., Kitchener, Ontario, Canada) (electronic supplementary material, data S1). Their ratios (emission acceptor (515 nm)/emission donor (410 nm)) [73] were box plotted against the respective PDF concentration (*n* = 8 each) in dose–response curves. The control experiments were conducted with cells expressing Epac-L alone (*n* = 4; −*He-pdfr-A/B* in figure 2*d*,*e*), stimulation without PDFs (*n* = 8; −PDF in figure 2*d*,*e*), and exposure to Forskolin (50 μM final concentration; Sigma-Aldrich) and IBMX (100 μM final concentration; Sigma-Aldrich), respectively (at least *n* = 3; +Forsk./IBMX in figure 2*d*,*e*). Relative EC_50_ values were estimated from dose–response data using the online version of Quest Graph™ EC_50_ Calculator (https://www.aatbio.com/tools/ec50-calculator) by conducting a nonlinear regression analysis using a sigmoidal four-parameter logistic model.

### 4.7. Synthesis of antibodies and specificity tests

In a previous study [21], specimens of *H. exemplaris* (formerly referred to as *H. dujardini* [40]) failed to stain with a polyclonal antiserum raised against the synthetic β-PDH of the crustacean *Uca pugilator* (code 3B3; Dircksen *et al*. [24]; catalogue no. PDH beta; RRID: AB_231509; figure 2*b*), which has been widely used to localize PDHs/PDFs in a variety of animals [4,8,21,24,30]. We therefore generated customized polyclonal antibodies against each of the three mature PDFs of *H. exemplaris* as described previously for the onychophoran *Euperipatoides rowelli* [20]. The antibodies (anti-He-PDF-1: IG-P1037, LOT no. 2080B1, 25 µg ml^−1^; anti-He-PDF-2: IG-P1038, LOT no. 2025B2, 22 µg ml^−1^; and anti-He-PDF-3: IG-P1039, LOT no. 2082B, 31 µg ml^−1^) (electronic supplementary material, table S4) were commercially produced and purified (immunoGlobe GmbH, Himmelstadt, Germany) as described previously [20]. In brief, polyclonal antibodies against He-PDF-1, He-PDF-2 and He-PDF-3 were produced by using HPLC-purified synthetic He-PDF-1 (NAEVLNSLIGLPRLLKDK-NH_2_), He-PDF-2 (NSEILNTIIGLPNKLRQR-NH_2_) and He-PDF-3 peptides (NSEILNTLIGLPNKLKQR-NH_2_; peptides&elephants GmbH (Hennigsdorf, Germany). Before immunization in rabbits, the peptides were conjugated with Sulfo-SMCC-activated bovine thyroglobulin (Merck) as carrier. By using a twofold depletion method, three anti-He-PDF antibodies were purified from the sera of immunized rabbits, and to further reduce cross-reactivity, affinity purification was performed with its own antigen (immunoGlobe GmbH, Himmelstadt, Germany). To clarify the spatial relationship between parts of the visual system (eye) and PDF-*ir* cells, we further generated a specific antibody against the visual rhabdomeric opsin of *H. exemplaris* (anti-He-R-Opsin-V: ID no. 78−01−15; 0.8 mg ml^−1^), based on the previously identified *r-opsin-v* sequence (formerly referred to as ‘*Hd-r-opsin Hypsibius dujardini*’ [74]; GenBank accession number: KM086335). The antibody was commercially synthetized and purified by Peptide Specialty Laboratories GmbH, Heidelberg, Germany (electronic supplementary material, table S4).

For testing the specificity of the customized antibodies as well as the previously used polyclonal antiserum against the β-PDH peptide of *Uca pugilator*, western blots were performed using synthetic He-PDF-1, He-PDF-2 and He-PDF-3 peptides (Biomatik Corp.). First, gradient acrylamide Tris/Tricine gels (4%−10%−16%) were prepared [75] and each well was loaded with 400 ng of the corresponding peptide, mixed with 2× Laemmli sample buffer (0.125 M Tris base, 0.14 M SDS, 20% glycerol, 10% 2-mercaptoethanol, Orange-G; pH 6.8). One well was loaded with 6 µl of a protein standard (Spectra™ Multicolor Low Range Protein Ladder; Thermo Fisher Scientific). The peptides were separated in the gels by SDS-PAGE and subsequently transferred to PVDF membranes with 0.2 μm pore size (Carl Roth GmbH & Co. KG, Karlsruhe, Germany) using the semi-dry western blot technique. The membranes were blocked with 4% bovine serum albumin (BSA; Carl Roth GmbH & Co. KG) in Tris-buffered saline containing Tween 20 (TBS-T; 0.02 M Tris base, 0,15 M sodium chloride, 0.05% Tween 20; pH 7.4) for 60 min and incubated with primary antibodies (anti-He-PDF-1: 1 µg ml^−1^; anti-He-PDF-2: 1 µg ml^−1^; anti-He-PDF-3: 1.5 µg ml^−1^; diluted in TBS-T containing 1% BSA) overnight at 4°C. On the following day, after a few rinses with TBS-T, the membranes were incubated with peroxidase-conjugated goat anti-rabbit IgG (1 : 5000; Jackson ImmunoResearch, West Grove, PA, USA) at room temperature for 3 h. Following multiple rinses with TBS-T, a chemiluminescent substrate (Pierce™ ECL Western Blotting Substrate; Thermo Fisher Scientific) was added prior to detection. Finally, the Odyssey^®^ Fc Imager (LI-COR Biosciences, NE, USA) was used for imaging the membranes. Brightness and contrast of acquired images were adjusted using Image Studio Version 6.0.0.28 (LI-COR Biosciences).

We performed additional sets of western blot experiments using lysates from several hundred specimens of *H. exemplaris* to detect endogenous He-PDF-1, as this is the most abundant PDF peptide. However, we could not detect a specific signal because of a prominent green stain on the PVDF membrane, which was most likely due to the algae from the tardigrade gut. This stain appeared in the same region where the PDF bands were expected, and it strongly interfered with the chemiluminescence detection of the HRP-conjugated secondary antibody. Second, we performed loading controls using several methods (Ponceau, Coomassie and silver staining), but none of these detection methods revealed protein bands in any lane. Nevertheless, we consider the results of our immunolabelling experiments (figures 3*a*,*c*,*e*, 4*a*–*o* and 5*a*; electronic supplementary material, figures S5, S6*a–c* and S9*a*,*d*) are valid, as they are consistent with our mRNA expression data (figures 3*b*,*d*, 5*b*, 6*a*–*c*; electronic supplementary material, figures S6*d,e*, S8 and S9*b*,*e*) on the three PDF peptides of *H. exemplaris*.

### 4.8. Immunohistochemistry

The preparation of specimens for immunohistochemistry, including asphyxiation, puncturing and enzyme treatment, was performed as described previously [60], except that we used a different fixative [76] (4% PFA, 7.5% picric acid in 0.1 M PBS, pH 7.4) for fixation. After several rinses with PBS-Tx (PBS, 1% Triton X100), specimens were incubated in blocking solution containing 10% normal goat serum (NGS; Sigma-Aldrich) in PBS-Tx for 1 h at 4°C. Thereafter, primary antibodies (either rabbit anti-He-PDF-1 (0.125 µg ml^−1^ in PBS-Tx, 1% NGS), rabbit anti-He-PDF-2 (1 µg ml^−1^ in PBS-Tx, 1% NGS) or rabbit anti-He-PDF-3 (3.1 µg ml^−1^ in PBS-Tx, 1% NGS)) were applied and incubated for 3 days at 4°C. Following multiple washes in PBS-Tx, a fluorescently conjugated secondary antibody (either goat anti-rabbit Alexa Fluor^®^ 488 (1:2000; Thermo Fisher Scientific) or goat anti-rabbit Alexa Fluor^®^ 568 (1:2000; Thermo Fisher Scientific)) were applied for 2 days at 4°C (electronic supplementary material, table S5). After several rinses with PBS-Tx, specimens were incubated in a solution containing the DNA marker DAPI (4′,6-diamidino-2-phenylindole; 1 ng ml^−1^; Carl Roth GmbH & Co. KG) for 1 h at room temperature and then mounted in ProLong™ Gold Antifade Mountant (Thermo Fisher Scientific) between two cover slips.

For double immunolabelling, two sets of experiments were conducted, combining either rabbit He-PDF-1 with rabbit He-PDF-2 or rabbit He-PDF-1 with rabbit He-PDF-3 antibodies, respectively. Beyond these modifications, we followed a previous protocol [20] for double immunolabelling. Affinipure Fab Fragments (goat anti-rabbit; 40 μg ml^−1^; Jackson ImmunoResearch) in a solution of PBS-Tx with 1% bovine serum albumin (BSA) were used for double immunolabelling to distinguish each two PDFs (electronic supplementary material, table S6). The same concentrations of primary antibodies were used as for single immunolabelling and incubation proceeded for 2 days. We further performed double immunolabelling with the He-R-Opsin-V (10 µg ml^−1^ in PBS-Tx, 1% NGS) and the He-PDF-1 (0.125 µg ml^−1^ in PBS-Tx, 1% NGS) antibodies (electronic supplementary material, table S4), using both simultaneously. The methodology was the same as for single immunolabelling.

### 4.9. *In situ* hybridization

For mRNA detection using hybridization chain reaction–fluorescence *in situ* hybridization (HCR-FISH), split-initiator probes for *He-pdf-1*, *He-pdf-2*, *He-pdf-3, He-pdfr* and *He-r-opsin-v* were synthesized by Molecular Instruments, Los Angeles, CA, USA (electronic supplementary material, table S1). We followed a previous protocol [77] for preparing the specimens of *H. exemplaris* for conducting HCR-FISH with the following alterations: asphyxiated specimens were fixed in 4% PFA (in PBS, pH 7.4) for 1.5 h. Fixed specimens were incubated with Proteinase K (0.25 mg ml^−1^ in PBS-Tw (PBS with 1% Tween 20); Thermo Fisher Scientific) for 30 min at room temperature. Thereafter, HCR-FISH was performed as described previously [78] with the following modifications: fixed specimens were first incubated with the probes for 3 days at 37°C and then with the amplifier reagent conjugated to a fluorophore for 2 days at room temperature. The specimens were counterstained with DAPI for 1 h, rinsed several times in PBS and mounted between two cover slips in ProLong™ Gold antifade Reagent (Thermo Fisher Scientific). Double HCR-FISH was conducted by simultaneously applying two probes. Double labelling of mRNA and the respective protein/peptide was performed by combining HCR-FISH with immunostaining. To achieve this, first protein detection and then mRNA detection were performed following a previous protocol [78] with the following modification: the incubation with the initiator-labelled secondary antibodies was carried out overnight. Negative control experiments were performed by leaving out the probes but adding the respective amplifiers. Positive control experiments were conducted by using sets of redundant probes [79] synthesized by Molecular Instruments.

In addition to HCR-FISH, we performed classical whole-mount *in situ* hybridization using digoxigenin-labelled RNA probes (*He-pdf-1*, *He-pdf-2* and *He-pdf-3*; 1−3 ng μl^−1^ each) according to a previous protocol [77] with the same modifications described above for HCR-FISH. Hybridization was carried for 3 days at 60−63°C. Anti-DIG-AP Fab fragments antibody (diluted 1 : 4000 in 2% NGS in PBS-Tw, Roche, Mannheim, Germany) was applied for 3 days at 4°C. After several rinses with PBS-Tw, chromogenic staining was done either with NBT/BCIP (Thermo Fisher Scientific) solution or Fast Red Kit (Sigma-Aldrich) in the dark and the reaction was stopped when desired intensity of signal was achieved. Specimens were mounted in Fluoromount-G™ (Thermo Fisher Scientific) and imaged under a bright-field microscope.

### 4.10. Microscopy, three-dimensional reconstructions and panel design

Specimens were analysed using a confocal laser scanning microscope with an Airyscan module (Zeiss LSM 880; Carl Zeiss Microscopy GmbH). Raw Airyscan datasets were processed with the ZEN 2 imaging software (black edition; Carl Zeiss Microscopy GmbH). Brightness, contrast and sharpness of z-stack images were adjusted in Fiji v. 1.52 [80]. The LUT option was used in Fiji for assigning false colours to stained structures. Three-dimensional (3D) models were created based on selected high-resolution confocal datasets using a segmentation tool and volume rendering in Amira 6.0.1 (Thermo Fisher Scientific). Specimens labelled with the classical non-fluorescence chromogenic stain (NBT/BCIP and Fast Red Kit) were imaged under a bright-field microscope (Axio Imager.M2; Carl Zeiss Microscopy GmbH) equipped with a digital camera (Axiocam 503 color; Carl Zeiss Microscopy GmbH). Panels and diagrams were designed with Photoshop 24.5.0 and Illustrator 27.6.1 (Adobe Inc.).

## Data Availability

The original sources of transcriptomic and genomic assemblies used in this study are listed in the electronic supplementary material (table S1). The raw data used for this article will be made available by the authors on request. Sequenced clones of *He-pdfr-A* and *He-pdfr-B* were deposited in GenBank under the accession numbers PQ050056 and PQ050057. Supplementary material is available online [81].
